# Insights from quantitative analysis and mathematical modelling on the proposed WHO 2030 goals for soil-transmitted helminths

**DOI:** 10.12688/gatesopenres.13077.2

**Published:** 2019-12-04

**Authors:** NTD Modelling Consortium discussion group on soil-transmitted helminths

**Keywords:** Soil-transmitted helminths, WHO guidelines, morbidity control, NTD Modelling

## Abstract

Soil-transmitted helminths (STHs) are a group of parasitic worms that infect humans, causing a wide spectrum of disease, notably anaemia, growth retardation, and delayed cognitive development. The three main STHs are *Ascaris lumbricoides*, *Trichuris trichiura* and hookworm ( *Necator americanus* and *Ancylostoma duodenale*). Approximately 1.5 billion people are infected with STHs worldwide. The World Health Organization goal for 2030 is morbidity control, defined as reaching <2% prevalence of medium-to-high intensity infections in preschool-age children and school-age children (SAC). Treatment guidelines for achieving this goal have been recommended. The Neglected Tropical Diseases Modelling Consortium has developed mathematical and statistical models to quantify, predict, and evaluate the impact of control measures on STHs. These models show that the morbidity target can be achieved following current guidelines in moderate prevalence settings (20-50% in SAC). In high prevalence settings, semi-annual preventive chemotherapy (PC) ideally including adults, or at least women of reproductive age, is required. For *T. trichiura*, dual therapy with albendazole and ivermectin is required. In general, stopping PC is not possible without infection resurgence, unless effective measures for improved access to water, hygiene, and sanitation have been implemented, or elimination of transmission has been achieved. Current diagnostic methods are based on egg counts in stool samples, but these are known to have poor sensitivity at low prevalence levels. A target threshold for novel, more sensitive diagnostics should be defined relative to currently preferred diagnostics (Kato-Katz). Our analyses identify the extent of systematic non-access to treatment and the individual patterns of compliance over multiple rounds of treatment as the biggest unknowns and the main impediment to reaching the target. Moreover, the link between morbidity and infection intensity has not been fully elucidated. By providing more insights on all the above, we aim to inform discussions on the goals and treatment guidelines for STHs.

## Disclaimer

The views and opinions expressed in this article are those of the authors and do not necessarily reflect the views of the World Health Organization. Publication in Gates Open Research does not imply endorsement by the Gates Foundation.

## Background

Soil-transmitted helminth (STH) infections are caused by several species of parasitic worms that are transmitted by eggs present in human faeces, which contaminate the soil in areas with poor sanitation. STHs cause some of the most common infections, with about 1.5 billion people infected worldwide ^1^. The three main STHs are roundworm ( *Ascaris lumbricoides*), whipworm ( *Trichuris trichiura*) and hookworm ( *Necator americanus* and *Ancylostoma duodenale*). STHs reduce the nutritional status of infected individuals ^1^. In particular, infected children can be affected by reduced physical fitness and impaired growth and cognitive development ^1^. Hookworm infection in women of reproductive age (WRA) can lead to severe anaemia ^1^. Infections with *A. lumbricoides* and hookworms can be treated effectively with benzimidazole drugs (albendazole, mebendazole). However, benzimidazoles are less effective against *T. trichiura*. Dual treatment with albendazole and ivermectin increases treatment efficacy for *T. trichiura* ^2– 4^. Currently, albendazole and mebendazole are donated to the World Health Organization (WHO) for distribution to affected populations.

**Figure 1.**
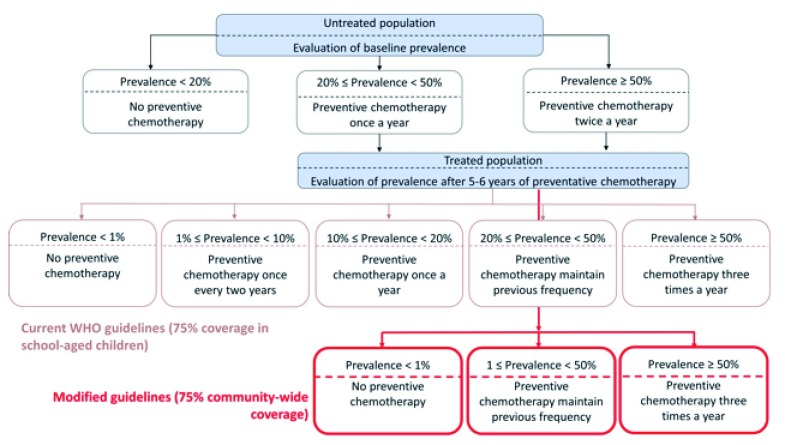
World Health Organization (WHO) decision tree showing the current WHO guidelines to achieve morbidity control in school-aged children (SAC) using 75% coverage in SAC (black and orange boxes). The bright red boxes represent the modified guidelines assuming 75% community-wide coverage (pre-SAC, SAC, and adults to replace the middle row of boxes that represent the current guidelines. This figure has been adapted from Figure 1 in 5 under a CC-BY 4.0 license.

The WHO has announced morbidity control as the main public health target for STHs to be achieved by 2030. According to the most recent WHO guidelines, morbidity control is defined as <2% prevalence of medium-to-high intensity (M&HI) infections in preschool-age children (preSAC) and school-age children (SAC). WHO treatment guidelines advise preventive chemotherapy (PC) by mass drug administration (MDA) to achieve morbidity control. Previously, WHO recommended school-based PC without including adults. The most recent guidelines recommend PC targeted at preSAC, SAC and WRA. The frequency of PC is based on the prevalence of STH infections in SAC prior to the start of treatment (see decision tree in Figure 1 for WHO guidelines up until 2019). The recommended PC coverage is 75% in all targeted populations.

Mathematical models of STH transmission dynamics and the impact of interventions have been developed to evaluate optimal treatment strategies for achieving the WHO goals. The Neglected Tropical Diseases Modelling Consortium (NTDmc) funded by the Bill and Melinda Gates Foundation brings together research groups from multiple scientific institutions working on neglected tropical diseases (NTDs), including STHs. Modelling groups based at Erasmus Medical Center (EMC) in Rotterdam and Imperial College London (ICL) have led the recent work on STHs. A model comparison was carried out for the EMC and ICL STH models ^6^. Moreover, joint papers evaluating WHO treatment guidelines and monitoring and evaluation strategies have been published ^5, 7^. Predictions have been made for each STH species to account for inter-species variability. In these predictions it is important to note that models predict true prevalence while surveillance data make predictions that are sensitive to the diagnostic method employed. The predictions of both models are largely comparable, although the EMC model is more optimistic about the additional impact of community-wide vs. targeted (pre-SAC, SAC and WRA) deworming, which can be readily explained by differences in assumptions about how rates at which individuals contaminate the environment vary with age.

The WHO has proposed new goals for NTDs, including new control targets for STHs in the year 2030. Using insights from recent modelling work we discuss the feasibility of reaching the morbidity target following current guidelines and the risks that need to be mitigated to maintain the target (summary in Table 1).

**Table 1.** Summary of modelling insights and challenges for reaching the WHO 2030 goal for soil-transmitted helminths.

Current WHO Goal (2020)	Morbidity control: <1% prevalence of M&HI infections in preSAC and SAC
Proposed new WHO Goal (2030)	Morbidity control: <2% prevalence of M&HI infections in preSAC and SAC
Is the new target technically feasible under the current disease strategy?	Yes, in moderate prevalence settings (20–50% in SAC) in the absence of systematic non-access to treatment. For highly endemic settings (prevalence ≥50%), semi-annual PC, including adults or at least WRA (hookworm), and/or dual PC (Trichuris) are required. A lot depends on the diagnostic used and these targets may have to be revised if the employment of PCR methods reveals much higher levels of infection.
Are current tools able to reliably measure the target?	Yes, although there is a need to test and identify the optimal design for surveys based on Kato-Katz (how many stool samples per person, how many slides per sample) and PCR for the specific purpose of evaluating the target and intermediate markers of progress (which are based on prevalence of any infection instead of M&HI).
What are the biggest unknowns?	Levels of systematic non-access or non-compliance to treatment and its impact on achievement of the target; the link between morbidity and present and past cumulated infection intensity and how the current parasitological target translates to actual morbidity levels; epidemiological situation in settings with pre- control prevalences <20% (meaning no implementation of PC) as PC in other areas continues.
What are the biggest risks?	Systematic non-access and non-compliance to treatment, low coverage and resurgence after reducing treatment frequency, lack of community-wide treatment, especially when hookworm is the dominant infection if the intrinsic transmission potential is high.

WHO, World Health Organization; M&HI, medium-to-high intensity; preSAC, preschool-age children; SAC, school-age children; PC, preventative chemotherapy; WRA, women of reproductive age; PCR, polymerase chain reaction.

## Insights from modelling: Lessons from the past 10 years for the next 10 years

Our modeling and epidemiological data analyses have shown that the current WHO treatment guidelines are sufficient to achieve the 2020 morbidity target in settings where the prevalence was moderate (20% to 50% in SAC) prior to the start of PC ^5^. For higher prevalence settings, community-wide PC and/or targeting of WRA will be necessary to achieve the morbidity target, especially for hookworm ^5, 8^, and/or dual therapy with albendazole and ivermectin for *T. trichiura* ^5, 9^. Implementing PC twice-yearly also increases chance to achieve the morbidity target for STH ^5^. Scaling down or stopping PC as per WHO treatment guidelines is very likely to lead to resurgence of infection to levels above the morbidity target, unless transmission conditions are addressed with water, sanitation and hygiene (WASH) ^10^ or elimination of transmission (EOT) is achieved ^5^. If this is not feasible, PC needs to be sustained ^10^. Impact assessments potentially need to be repeated at regular intervals. Accurate measurements of access and compliance to PC remain essential to evaluate and sustain achievement of the targets. It is also important to note the poor sensitivity of Kato-Katz at low prevalence (models predict true prevalence).

## Practical implications of the proposed goals

### Measuring the target

Geospatial analyses of recent large-scale epidemiological studies of STH prevalence ^11^ show that prevalence heterogeneity is considerable within PC implementation units. Our simulations suggest that to evaluate PC impact, a sufficient number of villages should be sampled in each implementation unit ^7^ for an accurate assessment of the prevalence (number of villages depending on geospatial variation).

The indicator for the morbidity target will be measured with Kato-Katz (or any other validated quantitative technique). However, the number of slides/samples used strongly affects the measured prevalence ^12, 13^. PCR methods, although expensive at present, are a much more sensitive diagnostic at low prevalence. The indicator threshold would be more meaningful if linked to a standardized diagnostic procedure, or to the true prevalence of infection from which thresholds for specific diagnostic procedures and sampling designs can be derived.

Current egg counting methods suffer from considerable measurement error, which is compounded by high variation in egg density between and within persons over time ^13– 15^, meaning that an observed prevalence of M&HI can be well above the 2% target by chance. This is further compounded by an increase in inter-individual variation in egg counts as infection prevalence goes down during PC (likely due to systematic non-access to PC). Further, modelling suggests that “prevalence of any infection in preSAC and SAC” combined with a higher target threshold is a more informative indicator (higher positive predictive value) for meeting the morbidity target and would require a smaller sample size because of a higher statistical power ^7^.

### Timeline to achieve the target

The 2030 morbidity target will be achievable in some countries. The frequency and duration of PC and implied resources depend on baseline prevalence and achieved coverage plus patterns of individual compliance to treatment ^5^.

### Technical feasibility

Treatment guidelines will lead to the achievement of the target in some communities, but not in all ^5^. Current WHO guidelines do not call for treatment in low prevalence settings (<20%). However, these areas may still have a prevalence of M&HI >2% in preSAC, SAC and WRA ^14^. In addition, epidemiological data from the Tumikia study suggest that with lower prevalence, the prevalence of M&HI is relatively higher due to increasing aggregation of parasites as MDA coverage rises, likely due to a small proportion of persistent non-compliers to treatment. A revision of the 20% threshold downwards seems desirable.

WHO guidelines for moderate-prevalence settings suggest annual PC of young children, preSAC, SAC and WRA. This may be sufficient to reach the morbidity target for settings where coverage is sufficiently high (75%) in the absence of systematic non-compliance to treatment.

For high-prevalence settings (>50%), WHO guidelines suggest semiannual treatment. Here the morbidity target is less likely to be achieved following current guidelines, especially for hookworm and *T. trichiura*. As the main burden of hookworm infection lies in adults, the morbidity targets will only be reached when also treating adults as a whole ^5^, not just WRA ^8^. Control of *T. trichiura* will require community-wide treatment with albendazole ^3^ or dual treatment with ivermectin and a benzimidazole ^3, 5^. If systematic non-access to treatment remains high, meeting the target may not be feasible ^8^.

### Operational feasibility

Reaching the milestone in 2030 will require community-wide coverage and/or targeting of WRA (especially for hookworm), with low systematic non-access/non-compliance to PC and little coverage heterogeneity within PC implementation units. Modelling suggests that the timeline for achieving the target is expected to be longer if there is re-importation of disease, e.g. by migration for areas with low or no treatment coverage ^16^. Meeting the target may require coordination of national STH programmes at country borders due to human movement.

### Ability to sustain achievement of the goal

After stopping or scaling down treatment (which is an option in the current WHO guidelines, see Figure 1), infection levels are likely to bounce back within one to two years ^17^. Thus, it may not be possible to decrease the number of required tablets as proposed as a new WHO target ^5, 8^. This is further complicated by population growth between now and 2030, which could necessitate a further increase in the number of treatments required for pre-SAC and SAC. See Figure 1 for an alternative decision-tree based on recent modelling.

Our analyses suggest that uptake of effective WASH is needed to sustain the gains made by PC in the longer term ^10^. If EOT is not achieved and PC is stopped or scaled down in the absence of effective WASH, the probability of resurgence is very high ^5^. In the absence of effective WASH interventions, the sustainability of the morbidity targets is undermined by human population movement unless PC is continued indefinitely ^16^.

### Considerations of cost

Child-targeted treatment for hookworm is cost-effective at reducing morbidity in children, even in high-transmission settings ^18, 19^. Community-wide treatment is predicted to be more cost-effective in the longer term with respect to the overall morbidity case-years prevented than child-targeted treatment, as the main hookworm disease burden lies in adults ^18, 19^. Annual co-administration of albendazole/mebendazole with ivermectin is predicted to be more cost-effective than semi-annual albendazole/mebendazole treatment for reducing the prevalence of heavy *T. trichiura* infections in SAC ^9^. In general, achieving high coverage and good individual compliance in annual treatment rounds may be more cost-effective than treating twice a year with lower coverage.

## Risks that need to be mitigated to achieve the stated goals

Population movement can re-import infection into a geographical area that has previously reached morbidity control or EOT. Measures to mitigate this risk include aiming for evenly high coverage across implementation units and co-ordination of programmes across country borders ^16^.

Systematic non-access and non-compliance to treatment in repeated rounds of MDA and predisposition to heavy infection will create a pool of individuals with high infection burden that can re-infect others ^8, 10, 20– 24^. Increasing access as well as coverage will be important for achieving the 2030 targets ^22, 23^.

Reducing frequency of treatment, as proposed in WHO guidelines ^25^ and the new goal for 2030 of reducing the number of tablets required for treating STHs, in the absence of EOT and/or effective WASH measures (including both measures reducing contamination and measures reducing exposure to infection) can lead to rapid resurgence of infection prevalences to pre-treatment levels ^5, 10, 26– 28^. See Figure 1 for an alternative decision-tree based on recent model-based analyses.

## Discussion

The morbidity target is defined in terms of the prevalence of M&HI. However, infection intensity does not necessarily reflect morbidity accurately, as light infections can be associated with non-negligible morbidity and the severity of symptoms associated with M&HI is highly variable ^29^. Furthermore, current diagnostic tools have poor sensitivity at low prevalence levels. Defining targets depending on the diagnostic used seems desirable in future policy formulation.

Transmission dynamic models with parameters estimated from cross-sectional and longitudinal epidemiological data show that technically EOT is feasible for STH in some settings. It is predicted that EOT can be achieved in low-transmission settings where *A. lumbricoides* or *T. trichiura* are the dominant parasites by annual treatment of SAC, assuming 80% effective coverage and random compliance at each round of treatment ^30^. Where EOT is feasible, it may be more cost-effective than continuous morbidity control, provided no re-importation occurs ^19^. In high transmission settings, community-wide treatment is predicted to be more effective (especially for hookworm) and more cost-effective.

Another new WHO goal for 2030 is control of strongyloidiasis. This requires ivermectin treatment, which would particularly benefit areas with high prevalence of *T. trichiura*. Currently, for policy assessments there is only epidemiological data on *Strongyloides stercoralis* ^31, 32^ but no model-based predictions. As for the other STH, models will provide useful insights for policy formulation.

## Future work

Future work that the NTDmc can contribute in support of the design and achievement of the WHO 2030 goals will focus on: 1) an analysis of the value of different diagnostic methods and sampling strategies on M&E of STH morbidity targets and predicting the probability of EOT; 2) understanding the role of spatial heterogeneity in prevalence and coverage and human population movement on STH control programmes; 3) investigating the risk of emergence of drug resistance as well as whether and how monitoring of drug efficacy may help, 4) quantifying the link between infection intensity and morbidity; and 5) assessing the importance of different patterns of individual compliance to treatment to achieving the WHO targets as data becomes available from large-scale epidemiological studies and trials. Other proposed topics for future work include the impact of discontinuation of lymphatic filariasis programmes on STH, infection models encapsulating molecular epidemiology data of who infects whom, defining threshold values for when systematic non-access and non-compliance causes failure to achieve WHO targets, and development of transmission models for *Strongyloides stercoralis*.

## Data availability

No data are associated with this article.
