# Supplementary material for: Insights from quantitative analysis and mathematical modelling on the proposed WHO 2030 goals for soil-transmitted helminths
Source: Gates Open Res. 2019 Dec 4;3:1632. Originally published 2019 Oct 23. [Version 2] doi: 10.12688/gatesopenres.13077.2 (PMC6869437; doi:10.12688/gatesopenres.13077.2)
Supplement: Peer review history

# Peer review: Reviewer response for version 1

**DOI:** 10.21956/gatesopenres.14216.r28134

**Published:** 2019-11-18

**Authors:** Suzy J. Campbell

**Affiliations:** 1 Evidence Action: Deworm the World Initiative, Washington, DC, USA

The proposed World Health Organization (WHO) 2030 Roadmap goal of morbidity control for soil-transmitted helminths (STH) is defined as reaching <2% prevalence of moderate-to-heavy intensity infections (MHII) in preschool (PSAC) and school age children (SAC). In this manuscript, the NTD Modelling Consortium discussion group on STH provide a summary of insights from mathematical and statistical models into whether this goal is feasible to achieve, and to evaluate the optimal treatment strategies to reach it. In summary, based on two separate but comparable predictive models, the authors determine that the 2030 morbidity target could be achieved using current treatment guidelines in moderate settings (being those with 20-50% prevalence in SAC). Higher prevalence settings are deemed likely to require semi-annual preventive chemotherapy, ideally by including adults (or at least women of reproductive age, WRA), and combined albendazole-ivermectin against *Trichuris trichiura*. To prevent resurgence, preventive chemotherapy needs to be sustained, unless there are effective measures to improve access to water, sanitation and hygiene (WASH), or unless elimination of transmission (EOT) has been achieved. A target threshold for novel, sensitive diagnostic tests is required. The biggest unknowns, and greatest impediments to the target, are deemed to be systematic non-access to treatment and individual compliance over multiple rounds of mass drug administration (MDA). The authors recommend changes to the WHO decision trees, assuming 75% community-wide coverage. 

 Gaining this level of insight from the NTD Modelling Consortium is very useful and the article itself is well constructed. However, by modelling what would be required to reach the proposed 2030 goal, the authors highlight some key anomalies between the 2030 goal and the current international landscape. The first is that although WRA are not explicitly included in the goal definition, actions to achieve the goal for PSAC and SAC will require treatment of WRA, if not all adults, in certain high prevalence settings. There remains insufficient evidence at this time, particularly on cost-effectiveness, to scale up treatment of the WRA cohort via MDA campaigns. Further, in Figure 1, the authors suggest modified guidelines that assume 75% community-wide coverage. Achieving and sustaining 75% full community coverage would be very hard, and this is neither seen as particularly scalable nor necessary for a morbidity-control goal. WHO guidance is recommended to at-risk (targeted) populations and they stop short of recommending full community treatments. As is known, drug donations and/or implementation advice to reach expanded cohorts are not provided, and examples of programmatic expansion are rare. Is the authors’ recommendation of 75% community-wide coverage applicable to all settings, or has this extended beyond what is required for the current morbidity control goal?

 The authors are kindly requested to elucidate further on the statement (Discussion) that “it is predicted that EOT can be achieved in low-transmission settings where *Ascaris lumbricoides* or *T. trichiura* are the dominant parasites by annual treatment of SAC”. If this is the case, but this manuscript has investigated achieving the goal for scenarios above 20%, it seems unlikely that EOT would enter into consideration and thus, preventive chemotherapy would have to continue. The STH morbidity control goal is to eliminate heavy burden of disease, not eliminate a parasite. Whilst, biologically, EOT is feasible, this is not yet considered the best use of sparse resources, and it is an investment with a number of unknown factors that most countries would be inadequately prepared for.

 If neither WASH access nor EOT are feasible, then preventive chemotherapy needs to be sustained. This recommendation for indefinite preventive chemotherapy warrants further projections, for example to a second impact assessment, to determine for how long this may need to be the case. Potentially there could be recommendations for a second assessment layer to the decision trees (e.g., hypothetically after 10 years of MDA). For many countries, reaching the point of a second impact assessment will be reached within the 2020-2030 time period.

 The authors recommend co-administered albendazole-ivermectin against *T. trichiura*, and also community-wide treatment when hookworm is the dominant infection. This differentiation of some of the STH is welcomed, but WHO guidelines are for undifferentiated STH. Can the authors please advise what level of *T. trichiura* prevalence/intensity would be the recommended ‘trigger’ for co-administration? What level of hookworm prevalence/intensity would be the trigger for risk mitigation activities such as expanding treatments to additional cohorts? Were helminth-specific cutpoints considered in the modelling to arrive at the optimum treatment requirements? If not, is there intent to research this further, with the aim of providing additional advice for implementation considerations? Similarly, can the authors please indicate what aspects of “effective WASH” were modelled – was this access to a latrine, access to a water source, combined interventions, etc?

 What is concerning is that the insights from the NTD Modelling Consortium highlight that this new Roadmap goal will require more investment, and more drugs, for STH, but donations for both are likely to decrease. The clarion call from this manuscript is that unless there is careful long-term sustainability and alternative delivery planning, it could well be that the 2030 STH goal will be too ambitious for many settings.

I confirm that I have read this submission and believe that I have an appropriate level of expertise to confirm that it is of an acceptable scientific standard.

# Response

**Published:** 2019-12-02

**Authors:** Luc Edgar Coffeng

**Reviewer 2: **Is the authors’ recommendation of 75% community-wide coverage applicable to all settings, or has this extended beyond what is required for the current morbidity control goal?

**Answer: **As mentioned in the paper, community-wide coverage of 75% is especially important for settings where hookworm is the dominant STH. This is because of the age-prevalence profile of hookworm where adults bear the main burden of infection. In settings where *A. lumbricoides* or *T. trichiura* are the dominant species 75% coverage in adults is probably not strictly necessary provided that the coverage in pre-SAC and SAC is sufficiently high, because the age-prevalence profile peaks in children. We have not run simulations to determine the minimum PC coverage of adults required to reach the morbidity target in settings where *A. lumbricoides* or *T. trichiura* are the dominant species, given that 75% coverage of preSAC and SAC is achieved. The minimum PC coverage of adults required in these settings will depend on the baseline prevalence and transmission intensity.

**Reviewer 2:** The authors are kindly requested to elucidate further on the statement (Discussion) that “it is predicted that EOT can be achieved in low-transmission settings where *Ascaris lumbricoides* or *T. trichiura* are the dominant parasites by annual treatment of SAC”. If this is the case, but this manuscript has investigated achieving the goal for scenarios above 20%, it seems unlikely that EOT would enter into consideration and thus, preventive chemotherapy would have to continue.

**Answer:** The conclusion that EOT can be reached in low-transmission settings comes from earlier simulation studies that classified transmission settings using the basic reproductive number R _0,_ rather than baseline prevalence (1). Any given value of R _0_ does not directly translate into a particular prevalence of infection, because the prevalence also depends on other model parameters, such as the aggregation parameter k (describes the tendency that a few individuals will be infected by many worms while most individuals will have low-intensity infections) and the density-dependence of female worm fecundity. However, in general, low-prevalence settings will also have low transmission intensities (low R _0_) values. Therefore, one can predict that EOT can be achieved in low-transmission/low-prevalence settings. Moreover, it is possible that some moderate-prevalence settings also have low-transmission intensity as measured by R _0_. This needs to be estimated on a case-by-case basis. In these settings, EOT can also be achieved.

It is true that in settings where EOT cannot be achieved using current MDA strategies (all high-prevalence settings and some moderate-prevalence settings) and WASH facilities are not improved to the degree that transmission ceases, MDA would have to be continued indefinitely.

**Reviewer 2:** If neither WASH access nor EOT are feasible, then preventive chemotherapy needs to be sustained. This recommendation for indefinite preventive chemotherapy warrants further projections, for example to a second impact assessment, to determine for how long this may need to be the case. Potentially there could be recommendations for a second assessment layer to the decision trees (e.g., hypothetically after 10 years of MDA).

**Answer:** We agree with Reviewer 2 that repeated impact assessments are necessary where EOT cannot be achieved by MDA and/or WASH measures.

**Reviewer 2:** The authors recommend co-administered albendazole-ivermectin against *T. trichiura*, and also community-wide treatment when hookworm is the dominant infection. This differentiation of some of the STH is welcomed, but WHO guidelines are for undifferentiated STH. Can the authors please advise what level of *T. trichiura* prevalence/intensity would be the recommended ‘trigger’ for co-administration? What level of hookworm prevalence/intensity would be the trigger for risk mitigation activities such as expanding treatments to additional cohorts? Were helminth-specific cutpoints considered in the modelling to arrive at the optimum treatment requirements?

**Answer:** In the simulation models, cut-off points were investigated for each helminth species separately. We did not investigate settings where several STH species are present simultaneously. However, we would expect that cut-off values for one species would still be valid, regardless for the presence of other STH species. It follows that co-administration of albendazole-ivermectin in the case of *T. trichiura* or community-wide treatment in the case of hookworm are recommended when the prevalence of either species is above 20%. We have not investigated if intensified PC strategies are necessary where species-specific prevalences are lower.

**Reviewer 2**: Similarly, can the authors please indicate what aspects of “effective WASH” were modelled – was this access to a latrine, access to a water source, combined interventions, etc?

**Answer:** The WASH simulation study (2) distinguished two WASH modalities: sanitation, which reduces individuals’ contributions to environmental contamination, for example, access to latrines; and hygiene, which reduces individuals’ exposure to infection, for example hand washing, access to clean water sources. Both sanitation and hygiene measures in isolation and in combination were investigated. One interesting finding was that the impact of hygiene is determined more by the effectiveness of the intervention than its overall uptake, whereas the impact of sanitation depends more directly on the product of uptake and the effectiveness.

1.         Truscott JE, Hollingsworth TD, Brooker SJ, Anderson RM. Can chemotherapy alone eliminate the transmission of soil transmitted helminths? Parasites & vectors. 2014;7:266.

2.         Coffeng LE, Vaz Nery S, Gray DJ, Bakker R, de Vlas SJ, Clements ACA. Predicted short and long-term impact of deworming and water, hygiene, and sanitation on transmission of soil-transmitted helminths. PLoS neglected tropical diseases. 2018;12(12):e0006758.

# Peer review: Reviewer response for version 1

**DOI:** 10.21956/gatesopenres.14216.r28131

**Published:** 2019-11-05

**Authors:** Rubina Imtiaz

**Affiliations:** 1 Children Without Worms, The Task Force for Global Health, Decatur, GA, USA

This is a very well written Open Letter which clearly presents the background on Soil Transmitted Helminths, their health effects on risk populations, the WHO goals and treatment guidelines. The authors then state the proposed new, 2030 goals for STH "morbidity control" defined by <2% prevalence of medium- to high-intensity infections in pre-school age and school age children. 

 Two mathematical models were used to assess the feasibility and relative efficiency of WHO-proposed end goals, in light of current diagnostic and program-decisional thresholds. Good news is that these models are largely comparable. Key outcomes and recommendations from the models are nicely summarized in Table 1. Both models predict that the new target is feasible in moderate prevalence settings (20-50% in SAC) in the absence of systematic non-access to PC. For highly endemic settings (prevalence > 50%), they recommend semi-annual PC including adults, and dual PC for Trichuris. Shortcomings of the current diagnostic of choice (Kato Katz) are well discussed with options for newer, more sensitive diagnostics open (e.g. PCR methods in low prevalence settings).

 It’s very good to see the recommendation to have the flexibility in current indicator thresholds by WHO to accommodate future, more sensitive tests (link indicator threshold to standardized diagnostic procedure or to the true infection prevalence from which specific diagnostic procedures and sampling designs can be derived).  

 An additional consideration that may strengthen the model and recommendations for advanced programs, is to consider modeling for specific worms within the STH group: STH comprises 3 discrete parasites, each with its own different drug responsiveness and life cycle (e.g. hookworms have a shorter environmental life cycle, so more prone to "elimination" if adult worm reservoir is effectively reduced through sustained, high-level treatment cycles). So, the baseline data on species-specific burden of STH in an area, and appropriate treatment would be ideal. 

 In the absence of large scale quality data on STH morbidity, this article addresses the current issues very competently with some feasible recommended actions.

I confirm that I have read this submission and believe that I have an appropriate level of expertise to confirm that it is of an acceptable scientific standard.
